# “Assessment of the social influence and facilitating conditions that support nurses’ adoption of hospital electronic information management systems (HEIMS) in Ghana using the unified theory of acceptance and use of technology (UTAUT) model”

**DOI:** 10.1186/s12911-019-0956-z

**Published:** 2019-11-21

**Authors:** Lu Lin Zhou, Joseph Owusu-Marfo, Henry Asante Antwi, Maxwell Opuni Antwi, Arielle Doris Tetgoum Kachie, Sabina Ampon-Wireko

**Affiliations:** 1 Center for Medical Insurance, Hospital Management and Health Policy Research, School of Management, Jiangsu University, 301 Xuefu Road, Zhenjiang, Jiangsu 212013 People’s Republic of China; 2 Department of Health Informatics/Health Information Management, College of Health and Well-Being, Kintampo Bono East, Ghana

**Keywords:** Social influence, Facilitating conditions, Health information management, Hospital information systems, Health technology, Electronic health/medical records, Nurses, Ghana

## Abstract

**Background:**

Hospital electronic information management systems (HEIMS) are widely used in Ghana, and hence its performance must be carefully assessed. Nurses as clinical health personnel are the largest cluster of hospital staff and are the pillar of healthcare delivery. Therefore, they play a crucial role in the adoption and assessment of HEIMSs in Ghana. This report sought to assess the “Social Influence” (SI) and “Facilitating Conditions” (FC) that support Nurses’ Acceptance of HEIMS in Ghana using the “Unified Theory of Acceptance and Use of Technology” (UTAUT) model.

**Methods:**

This study applied a non-experimental survey design. An electronic platform questionnaire on smartphones was used to collect data on 660 nurses. Statistically, AMOS Structural Equation Modelling (SEM) version 22.0 was employed to examine the research model.

**Results:**

“Behavioral Intention” (BI) to HEIMS use was significantly predicted by SI and FC (*p* < 0.001). Notably, both SI and FC had an influence on nurses’ use behavior (UB) with behavioral intention (BI) as the mediator, which explains a total of 42.1% variance in the intention of nurses to use HEIMS. Likewise, UB of HEIMS was also significantly predicted by SI (R^2^ = 43.2) and BI (R^2^ = 0.39.6) with both constructs explaining a total of 51.7% of the variance in nurses’ acceptance to use HEIMS.

**Conclusion:**

Nurses’ adoption of HEIMS in terms of the UB was influenced by SI and BI, whiles SI and FC had the strongest influence on BI (serving as mediator) of UB to adopt and use HEIMS among the nurses in Ghanaian hospitals.

## Background

Health care organizations around the world continue to invest in health information technologies (HITs) [1, 2]. However, it has been recognized that user acceptance is one of the urgent issues in the implementation and the management of HITs [2]. Ghanaian Nurses work at the frontline of the healthcare system in the country with access to vital records about the patient populace in Ghanaian hospitals, hence, their usage assessment is necessary. Hospitals have invested considerable assets to enhance information technology (IT) infrastructure and set up medical institution record systems to enhance medical nursing care, administration efficiency and effectiveness as well as meet the challenges of an increasing number of the competitive enterprise environment and altering healthcare policies [3]. Nursing care is primarily based on a procedure that entails assessment, diagnosis, effects and planning, implementation, as well as assessment of the care of the affected person, while the documentation in the health report ought to mirror this system [4]. Besides, these nursing services are emerging field that has been substantially influenced by health technology usage. In this regard, some knowledge of technology/computing literacy is, therefore prerequisite for a nursing career in many healthcare centres. It is reported that healthcare providers like nurses are established to resist technology [5]. Pertinently, the successful implementation of any mHealth and electronic health records (EHRs) critically depends on user acceptance [6]. The EHRs seeks to allow the execution of patient-care-related health facility functions, which consists of patient administration, medical institution monetary affairs, and legal affairs, amongst different issues. Actually, EHRs is an integrated records system that performs a key function in supporting medical institution affairs via the use of suitable healthcare IT. This report aims; 1. to determine the social influence of Nurses and its impact on usage behavior of hospital electronic information management systems (HEIMS). 2. to assess the facilitating conditions of the hospitals and behavioral intentions (*A user’s readiness to carry out a particular behavior)* of Nurses that have an influence on usage behavior of HEIMS. The hypotheses were as follows;

**H1:** Social influence (SI) (moderators; professional experience; gender; age and voluntariness) will positively influence on behavioral intentions (BI) to use HEIMS, **H2:** SI will have a direct influence on usage behavior (UB)., **H3:** Facilitating conditions (FC) (moderators; professional experience; gender; age and voluntariness) will have a significant influence on BI., **H4:** FC will have a direct positive influence on UB and **H5:** BI will have a significant positive influence on usage. In this report, all health information technologies and EHRs platforms are termed as “Hospital Electronic Information Management Systems” (HEIMS). This report assesses Nurses’ acceptance of the adoption of “Hospital Electronic Information Management Systems” (HEIMS) via application of the UTAUT model [7]. The reason for using nurses as the study population is because they work at the frontline of the clinical healthcare system in Ghana with access to essential patient data in Ghanaian hospitals [8].

### “The unified theory of acceptance and use of technology (UTAUT) Model”

**Fig. 1:**
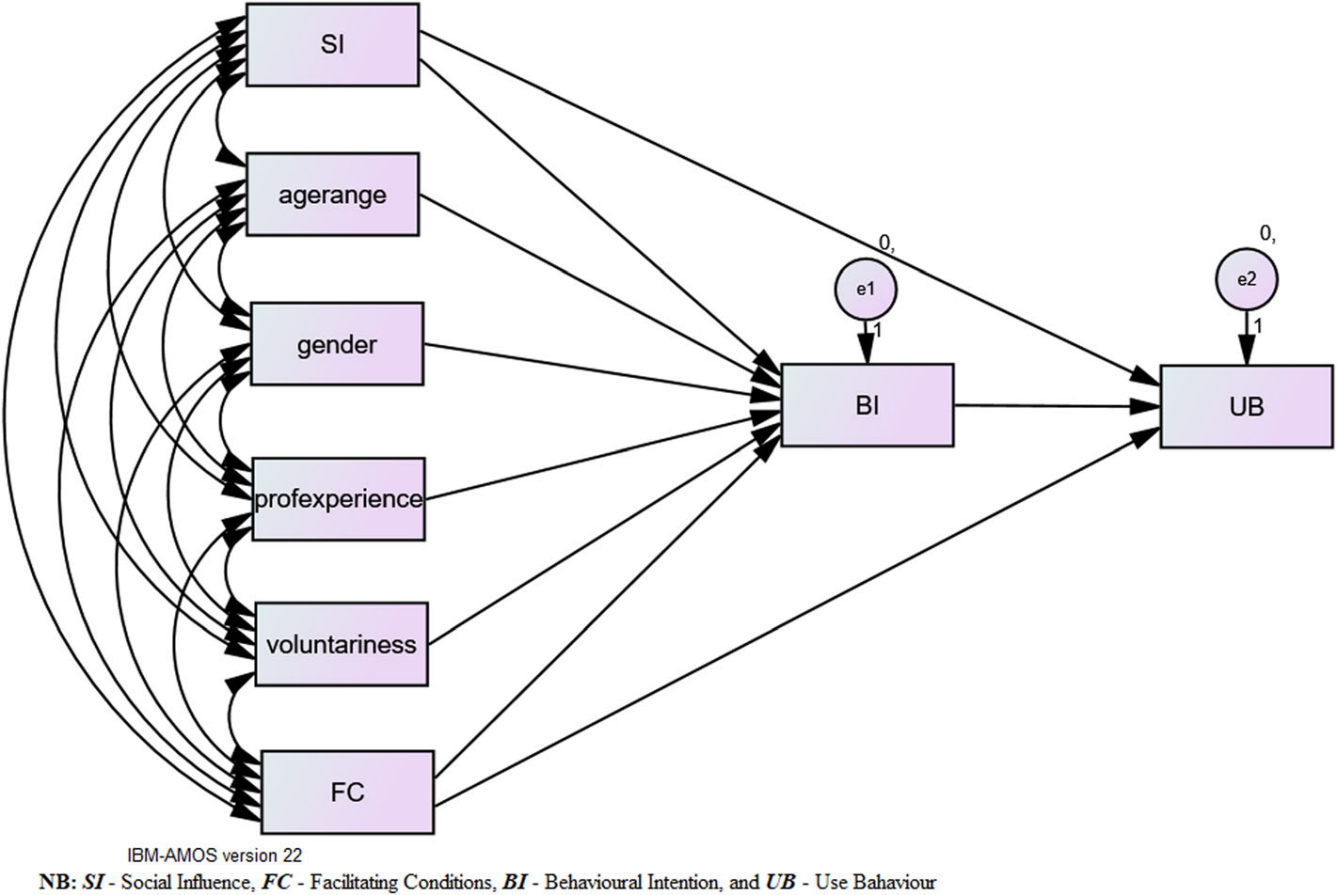
*Conceptual Model*

The proposed UTAUT model was validated appropriately to provide a unified theoretical groundwork for the promotion of research on information systems (IS) or adoption of IT and dissemination. Four (4) core parameters suggested by this model to directly determine health IT behavioral intentions and use behavior include performance expectancy, effort expectancy, social influence and facilitating conditions [7]. Additionally, the model assumed that the factors such as age, gender, experience and voluntariness to use greatly moderate the effect of key parameters [7]. The concept of UTAUT was developed via review, integration and mapping of eight dominant theories and models comprising; 1. “The Theory of Reasoned Action (TRA)”, 2. “the Technology Acceptance Model (TAM)”, 3. “the Motivational Model (MM)”, 4. “the Theory of Planned Behavior (TPB)”, 5. “a blended Theory of Planned Behavior/Technology Acceptance Model (C-TPB-TAM)”, 6. “the Model of PC Utilization (MPCU)”, 7. “the Innovation Diffusion Theory (IDT)”, and 8. “the Social Cognitive Theory (SCT)”. These models and theories have been efficaciously utilized through a variety of previous studies [7–10] on technology or adoption of innovation as well as circulation within both the ISs area and different disciplines including marketing, social health, psychology, and management science. Based on the argument that several of the concepts of the current theories are naturally comparable, UTAUT was motivationally defined and validated. In this context, it was, therefore, logical to map out and bring the various concept together and create a merged theoretical basis [7, 11, 12]. The proposed conceptual model adopted from the UTAUT model by [7] is depicted in (*Fig.* *1*).

## Methods

### Research study design, settings and participants

This study applied descriptive non-experimental survey study design. The research was conducted from January 15th to February 28th, 2019. This design was adopted in the present study because the researchers intended to assess the nurses use behavior of HEIMS at a point in time. This study focused on primary data derived from Nurses working in five major public hospitals in Ghana where HEIMS was being applied for patient care. All the Nurses who attended to patients care at the aforementioned hospitals qualified to participate in this study. Privately owned hospitals were excluded because they did not form part of the government’s EHRs (HEIMS) implementation plan at the time this study was conducted. A purposive sampling approach was used to select five public hospitals comprising of 3 teaching and 2 regional hospitals that use HEIMS in Ghana. Simple random sampling method was then used to recruit 660 nurses from the selected five government-owned hospitals.

### Research instrument

Numerical responses (form the Likert rating scale) were used in this survey via an electronic questionnaire to gather data from the respondents. Through preliminary study, UTAUT tool was established to be good enough for application in a cross-cultural investigation, like research involving participants from outside its original country of origin [13]. The UTAUT tool (particularly the original English version) was modified to fit the objectives of the present study [14]. The UTAUT constructs-related items in the questionnaire were ascertain using a 1 to 7-point Likert rating scale which ranged from “Strongly disagree (1)” to “Strongly agree (7)” for SI, FC and BI. Likewise, usage behavior was evaluated through a 1 to 8-point Likert rating scale that ranged from “Have not used (1)” to “Almost every day (8)”. Notably, extra information like age, gender, experience and voluntariness of use was obtained from the respondents using the questionnaire to serve as the moderators. The moderators were treated as categorical data for the analysis.

### Data collection procedure and statistical analysis

Data was collected using an organized questionnaire composing of items based on constructs validated in the original English version of the UTAUT model as described elsewhere [7]. An electronic data was obtained (via smart mobile phones and tablets) with structured questionnaire coded in Open Data Kit (ODK) application [15]. The questionnaire was pre-tested severally before being sent out for the data collection. The feedback of the pre-testing prompted the authors to make minor modifications to suit the study settings. The modified survey questions were tested for validity, reliability and accuracy. Almost all the selected nurses were approached during their working shifts and were contacted either through a mobile phone or verbally to take part in this study. The electronic questionnaire (made with ODK mobile application) were distributed to the nurses personally by the authors and other supporting data collectors. Completed questionnaires were automatically sent to our ODK google aggregate server-based website (www.odkproject-1333.appspot.com). All the questions were written in understandable English language and each questionnaire took about 4–10 min to complete. The questionnaire composed of forty-two questions including demographics.

Parameters were measured indirectly through indicators designed to elicit responses associated with a variable [16]. Most researchers use Structural Equation Modelling (SEM) as a measurement statistical tool for analysing data [17]. Statistically, SEM (IBM AMOS version 22.0) was employed to analyze the data that were collected. Actually, SEM is a multivariate approach that combines multiple regression factors and component analysis [16, 18]. In SEM evaluation, a causality network is estimated by researchers with respect to a theoretical model [19]. The main UTAUT study reported using the “partial least square” (PLS) path modeling approach [7] as having negligible demands on sampling size, residual or underlying data distributions, and measurement scales. Reliability is associated with the measurement within parameters which is independent of the state within other variables, while construct validity is associated with the measurement within parameters [20, 21]. The assessment of the “internal consistency reliability” (ICR) of parameters such as, “Cronbach’s alpha” (CA) was applied to examine the one-dimensionality of a group of the survey questionnaire measurements. Moreover, additional analysis such as “average variance extracted” (AVE) and “Composite reliability” (CR) for the individual construct provides coefficients which confirmed the reliability of the survey [22]. In this regard, 0.70, 0.70 and 0.50 were estimated as the respective recommended values [23]. The linear correlation coefficient (R^2^) of each dependent construct in the model was generated in order to assess whether the model fit well to the hypothesized relationship [24]. Notably, R^2^ is interpreted as the proportion of explained variance similar to regression analysis and therefore denotes the percentage of variance in the dependent constructs that is explained with the independent ones [25].

### Reliability and validity of measurement

The Cronbach’s Alpha for testing the overall reliability for the measurement items was 0.949, whiles the Kaiser-Meyer-Olkin Measure (KMO) of sampling adequacy was 0.928. The individual constructs validity and reliability testing results are shown in Table 3 and narrated in the results section.

## Results

### Descriptive statistics

This survey targeted 700 participants, but 660 respondents answered the questionnaire representing a response rate of 94.3%. Possibly, the innovative use of smart mobile phones might have ensured fast and easy answering of the questions, thereby resulting in a high response rate albeit participation being completely voluntary. Notably, the majority of nurses in Ghanaian hospitals are females. Hence, it was not surprising that 404 (61.2%) of the respondents were females. The respondents’ demographic data have been summarized and depicted in *Table* *1*. Staff nurse was the most usual nursing category title among the respondents (58.4%). Most respondents (52.4%) were between the ages of 20 and 30 years with all the respondents having at least a diploma degree (basic qualification to become a nurse in Ghana). A portion (48.03%) of the respondents had over one to five years of professional experience, and almost all the nurses have had at least one year of HEIMS experience. It was observed that all the respondents used two main types of HEIMS (lightwave health information management system (LHIMS) and hospital administration management system (HAMS)) in the regional and teaching hospitals in Ghana.

**Table 1:** Demographic Characteristics of Respondents

Parameter	Frequency(*N* = 660)	Percentage (%)
*1. Age of Respondents*		
20–30 years	346	52.4
31–40 years	251	38
41–50 years	63	9.6
*2****. Gender***		
Male	256	38.8
Female	404	61.2
*3. Professional Experience*		
0–5 years	317	48.03
6–10 years	163	24.70
11–15 years	92	13.94
16–20 years	76	11.52
21–25 years	12	1.81
*4.* ***Type of Nurse***		
Staff Nurse	386	58.4
Nursing Officer	206	31.2
Emergency Nurse	58	8.8
Critical Nurse	5	0.8
Pre-Operative Nurse	5	0.8
***5. Voluntariness***		
Completely not voluntary	173	26.2
Not Voluntary	328	49.7
Somewhat not voluntary	47	7.1
Neutral	31	4.7
Somewhat voluntary	31	4.7
Voluntary	43	6.5
Completely voluntary	7	1.1
***6. Type of HEIMS used***		
LHIMS	488	73.9
HAMS	172	26.1

***NB:*** *LHIMS – Lightwave Health Information Management Systems, HAMS – Hospital Administration Management Systems*

### The measurement models

The UTAUT model is composed of nine observed variable constructs. However, in this report, the measurement was primarily based on four of the constructs. The overall goodness of fit was assessed via some model fit measures. The results of the goodness of fit statistics are as follows; the Chi-square statistics (X^2^ = 42.78) to degrees of freedom (d.f. = 4) for the measured model [26]. The values of GFI (0.98), CFI (0.98), NFI (0.98), NNFI (0.96), and IFI (0.98) were greater-than 0.9 benchmark [26]. Likewise, the RMSEA was 0.06, which was within the acceptable levels of 0.05–0.08 [26]. As shown earlier, the required criteria for the majority of the fit indices were met as proposed by Hair et al. [26]. This result represents a reasonable fit between the obtained data and the suggested model measurement. In view of this, the instrument’s psychometric properties were evaluated regarding the “discriminant validity” (DV), “convergent validity” (CV) and reliability. The findings of the items’ loadings, means and the CA for all the measurement items (when an item is deleted) statistics are presented in Table 2.

**Table 2:** Result of Items Loading and Descriptive Statistics of Construct Measurements

Construct Measurements	Item loading λ	ε	Mean	Cronbach’s alpha (CA) (if item deleted)
PE1	0.880	0.226	5.760	.943
PE2	0.947	0.103	5.640	.943
PE3	0.928	0.139	5.730	.942
PE4	0.496	0.754	3.860	.946
EE1	0.914	0.165	5.540	.942
EE2	0.868	0.247	5.570	.942
EE3	0.932	0.131	5.640	.943
EE4	0.864	0.254	5.650	.943
ATUT1	0.592	0.650	5.660	.945
ATUT2	0.913	0.166	5.500	.942
ATUT3	0.893	0.203	5.230	.942
ATUT4	0.904	0.183	5.450	.942
SI1	0.913	0.166	5.700	.942
SI2	0.909	0.174	5.830	.942
SI3	0.280	0.922	3.870	.946
SI4	0.890	0.208	6.140	.943
SE1	0.842	0.290	5.400	.942
SE2	0.667	0.555	5.830	.944
SE3	0.904	0.182	5.150	.942
SE4	0.811	0.343	5.120	.943
FC1	0.550	0.698	5.530	.943
FC2	0.691	0.523	5.100	.943
FC3	0.843	0.289	2.900	.946
FC4	0.833	0.306	2.890	.947
ANX1	0.402	0.838	5.020	.943
ANX2	0.860	0.260	3.800	.948
ANX3	0.886	0.215	3.610	.948
ANX4	0.830	0.311	4.130	.947
BI1	0.985	0.030	5.590	.942
BI2	0.976	0.047	5.610	.942
BI3	0.980	0.040	5.580	.942
BI4	0.945	0.107	5.550	.943
UB1	0.949	0.099	7.820	.944
UB2	0.949	0.099	4.370	.942

***NB:*** *λ – factor loadings values, ε – error of the construct measurements values.* ***SI*** *– Social Influence,* ***FC*** *– Facilitating Conditions,* ***BI*** *– Behavioral Intention, and*

***UB*** *– Use Behavior*

Likewise, Table 3 displays the results of variances, CA, CR, AVE and DV as well as the Kaiser-Meyer-Olkin (KMO) measure of the sampling adequacy of the constructs. The CA for all the constructs was above the 0.7 thresholds (0.709 to 0.980). The assessment of the inner consistency of the measurement model was through the calculation of the CR of the constructs which ranged between 0.824 and 0.985 but exceeded the cut-off of 0.70 as recommended elsewhere [27]. In the current report, the construct validity was measured in terms of the usage of AVE and DV. Fornell and Larcker [28] proposed that the common AVE number exceeds 0.5 and is larger than every square correlation with recommended sufficient CV and DV. From Table 3 it could be observed that the AVE of the measurements ranged from 0.546 to 0.944, which surpassed the endorsed value (AVE > 0.5) [28]. This indicated a remarkable CV which is considered as one key criterion for acceptable DV [27]. Besides, it was discovered that all the AVE values were larger than every squared correlation coefficient, demonstrating the exact DV as indicated in Table 3 (the square root of AVE) and was proven with the aid of previous study [27]. The data obtained in this study had adequate CA, CR and DV. The sampling adequacy (KMO) of all the constructs were also higher than 0.5.

**Table 3:** Measurement Model; Results of AVE (Convergent Validity), CR, CA (Reliability Testing) and KMO (Sampling Adequacy Test)

Constructs	Mean	S. Dev.	Variance	AVE	CR	CA	KMO
PE	5.248	1.046	1.095	0.695	0.896	0.779	0.754
EE	5.600	0.934	0.873	0.801	0.941	0.915	0.802
ATUT	5.460	1.062	1.128	0.700	0.901	0.831	0.785
SI	5.386	1.078	1.163	0.633	0.859	0.709	0.745
SE	5.378	1.026	1.053	0.657	0.884	0.824	0.760
FC	4.105	1.183	1.399	0.546	0.824	0.723	0.564
ANX	4.140	1.256	1.577	0.594	0.845	0.753	0.708
BI	5.583	1.195	1.429	0.944	0.985	0.980	0.873
UB	6.094	1.978	3.912	0.901	0.948	0.842	0.560

NB: ***AVE*** *– Average Variance Extracted,* ***CR*** *– Composite Reliability,* ***CA*** *– Cronbach’s Alpha,* ***KMO*** *- Kaiser-Meyer-Olkin and* ***S. Dev*** *– Standard Deviation.* ***SI*** *– Social Influence,* ***FC*** *– Facilitating Conditions,* ***BI*** *– Behavioral Intention and* ***UB*** *– Use Behavior*

### The structural model

**Fig. 2:**
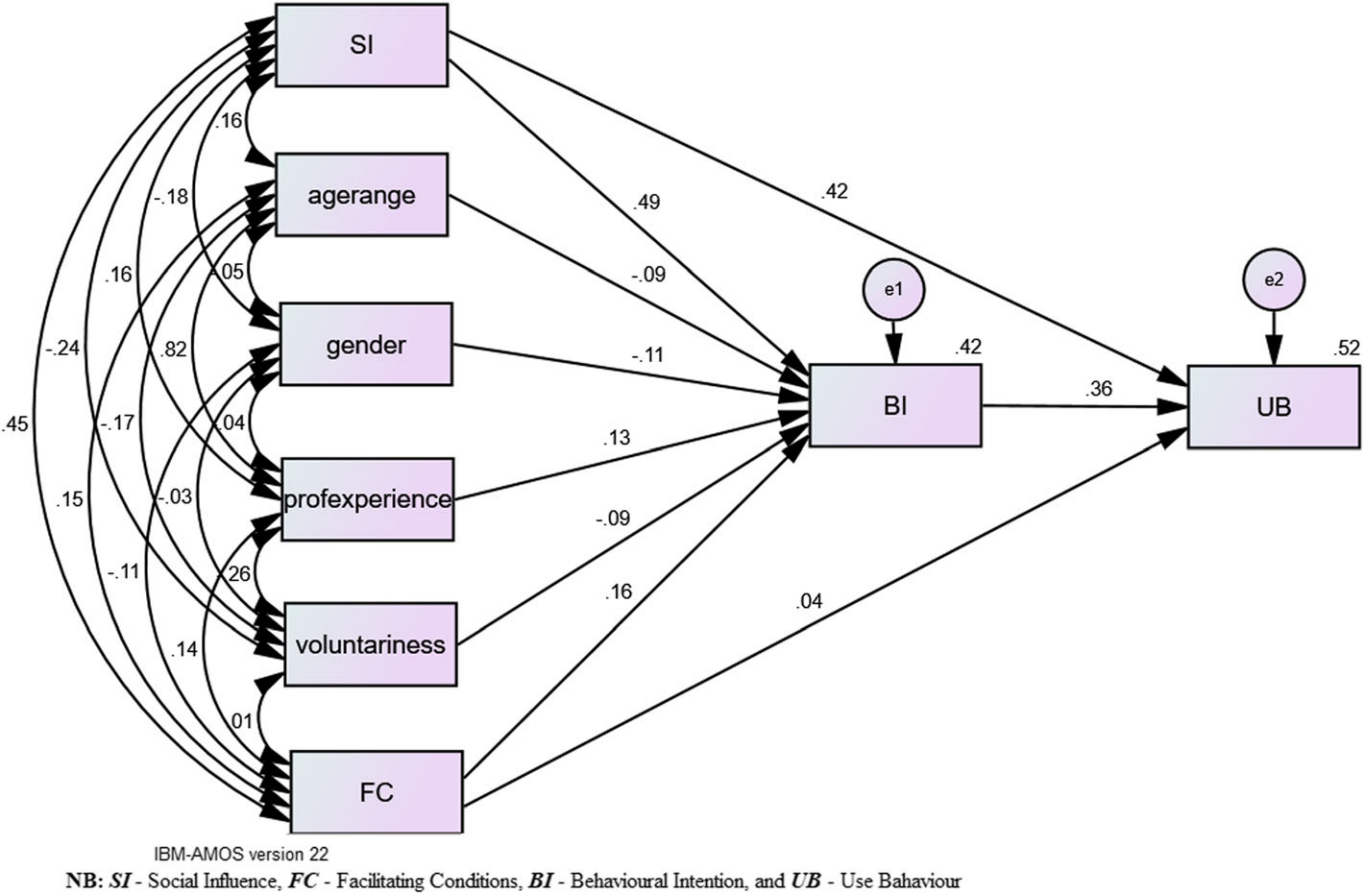
Standard Values of Structural Model

The structural model was examined via SEM technique with the effects of three latent variables tested. From Table 6; SI (age, prof. Experience, gender, and voluntariness acted as moderators) was explained by a variance of 40.1% (R^2^ = 0.401), while FC (moderated by age, prof. Experience, gender, and voluntariness) with just 24.8% (R^2^ = 0.248), and the model was wholly explained by a variance of 42.1% of BI (as mediator) to use HEIMS. In terms of usage behavior of HEIMS, SI and FC explained 43.2 and 14% of the variance respectively with BI explaining 39.6% of the variance while a total explained variance was 51.7%. *Fig.* *2* shows the standardized path coefficients which signify the substantial structural association within the tested parameters. The data showed that FC had no direct significant influence (*p* = 0.15) on the usage of HEIMS by the nurses, thus it did not support H4. Contrariwise, SI was shown to directly and significantly influence the usage behavior of nurses to use HEIMS (*p* < 0.001), thereby supporting H2. Furthermore, SI substantially impacted on the BI (as mediator) to use HEIMS (*p* < 0.001) and this supported H1. In addition, FC significantly influenced the usage behavior (*p* < 0.001) in a positive manner with BI playing the mediating role and thus, also supporting H3. Besides, BI predicted or had significance (*p* < 0.001) direct influence on UB of the nurses to use HEIMS, which also supported H5 (Table 4).

**Table 4:** Structural Model; Hypothesis Testing Results

Hypothesis	Parameter	R^2^	Estimate	S. E	C. R	Significance	Results
H1	BI <--- SI	0.38	.55	.04	14.06	***	Accepted
H2	UB < --- SI	0.43	.77	.07	11.76	***	Accepted
H3	BI <--- FC	0.16	.16	.03	4.80	***	Accepted
H4	UB < --- FC	0.14	.07	.05	1.44	0.15	Rejected
H5	UB < --- BI	0.40	.59	.06	10.22	***	Accepted

NB: ***p-value < 0.001, ***S.E****.- Standard Error,* ***C.R.*** *– Critical Ratio,* ***SI*** *– Social Influence,* ***FC*** *– Facilitating Conditions,* ***BI*** *– Behavioural Intention and* ***UB*** *– Use Behaviour. R*^*2*^ *– Square root of correlation coefficients of the intercepts*

## Discussion

The main findings of the current study are enumerated as follows: 1. SI and FC with BI serving as the mediator significantly influenced the UB of nurses to adopt and use HEIMS. 2. FC had an insignificant direct influence on UB to use the system. 3. Lastly, SI directly and significantly influenced UB and the adoption of HEIMS by nurses (Table 4 and Table 5). Nurses’ resistance to health technology use is high [5], however their intention to use HEIMS was observed to be encouraging and had significant influence. According to the description of SI from the UTAUT model, “it is peoples’ perception about how important they are to the society based on what they do or behave towards achieving their goals in their immediate working environment or surroundings” [13]. Image, social factors and subjective norms are, therefore, the three concepts of SI. The way people act or behave in their immediate environment is basically based on the aforementioned concepts which have substantial influence on what they do or not do. Most of the nurses’ adoption of HEIMS were based on these concepts. The BI was significantly influenced by SI to use and adopt HEIMS in Ghanaian hospitals. It is evident from the results (Table 4*)* that SI had significant influence on the intention of nurses to adopt and use HEIMS. In terms of Pearson’s correlation analysis, it was also evident that SI and BI had a positive significant correlation at *p* < 0.01 level (Table 5), which means that as the number of nurses with positive intention to use HEIMS increased, the number of nurses being influenced socially also increased. The SI also influenced UB directly towards the use of HEIMS (Table 4). With respect to the Pearson’s correlation analysis (Table 5), SI and UB had a positive significant correlation (p < 0.01), which also means that as SI on nurses increased with increasing UB towards the HEIMS adoption. Therefore, the hypotheses (H1 and H2) were accepted due to the significant results obtained after the testing. It could be concluded based on these results that most of the nurses’ reason that the use of the system will make them attain higher status than their colleagues who do not. It also suggests that the hospitals in Ghana might have encouraged and supported them to adopt HEIMS in the provision of quality healthcare services to their clients. UTAUT model description of FCs is the necessary IT resources to use a system and the technical infrastructure that exists to support this usage as well as the degree to which an individual believes that an organization can provide the necessary knowledge and training for HEIMS. Pertinently, since healthcare providers like nurses are likely to resist technology, there is the need to adequately equip them with technological equipment required for HEIMS usage and good training, else the adoption of any HEIMS could be rejected. The influence of FC with BI as the mediator on UB to use and adopt HEIMS was not statistically significant (Table 4 and *Fig.* *2*). The structural model standardized estimates and their probability values are usually known (as *p*-values) showed that FC did not reach statistical significance level. In view of this, the direct influence of FC on UB to use HEIMS hypothesis was rejected. Notwithstanding, FC recorded a strong significant relationship with UB (Table 5) which indicates that when nurses receive adequate training, they will be confident to accept and use new and advanced health technologies in future. The Pearson correlation between FC and BI was statistically significant (*p* < 0.01). From Table 4, it could be deduced that the results of the structural model of FC showed a significant positive influence on BI resulting in the acceptance of the hypothesis. The direct influence of BI on UB to use HEIMS was significant (*Table 4*) with explained 39.6% of variance associating with UB to use HEIMS. The hypothesis that BI will directly influence UB to use or adopt HEIMS was, therefore, accepted. Wholly, SI explained 43.2% of the variance associated with UB. Hence, SI, FC and BI all directly explained 51.7% of the variance associated with usage behavior (Table 6 and *Fig.* *2*).

**Table 5:** Partial Correlation Matrix of Constructs

Measure	Age	Gender	Prof. Experience	Vol	SI	FC	BI	UB
Age	**NA**							
Gender	−.051	**NA**						
Prof. Experience	.818^**^	−.043	**NA**					
Vol	.165**	.033	−.259**	**NA**				
SI	.157**	−.176**	.162**	−.244**	**0.796**			
FC	.147**	−.110**	.143**	.008	.448**	**0.739**		
BI	.135^**^	−.217^**^	.184^**^	−.219**	.612**	.399**	**0.972**	
UB	.200^**^	−.159^**^	.264^**^	−.316**	.657**	.375**	.630^**^	**0.949**

NB: **Correlation is significant at p-values < 0.01, the bold values on the leading diagonal show the discriminant validity (DV) of the constructs and the non-diagonal values are the correlation coefficients between the constructs. ***SI*** – Social Influence, ***FC*** – Facilitating Conditions, ***BI*** – Behavioural Intention, ***UB*** – Use Behaviour, ***Vol*** - Voluntariness

**Table 6:** Explained Variance of Constructs

Independent Variables (IV)		Dependent Variables (DV)	R-squared (R^2^)
Constructs	Moderators		
SI	no	BI	**0.375**
SI	age, gender, prof. Exp. & Vol.		**0.401**
FC	no		**0.160**
FC	age, gender, prof. Exp. & Vol.		**0.248**
SI & FC	age, gender, prof. Exp. & Vol.		**0.421**
SI	na	UB	**0.432**
FC	na		**0.140**
BI	na		**0.396**
SI, FC & BI	na		**0.517**

*R*^*2*^ *is the amount of variance in the dependent variable (****DV****) that is accounted for or explained by the independent variable(s) (****IV****).* ***BI*** *– Behavioural Intention,* ***UB*** *– Use Behaviour,* ***SI*** *– Social Influence and* ***FC*** *– Facilitating Conditions.* ***na*** *– not applicable,* ***Vol*** *- Voluntariness*

## Limitations

The outcome of this research is subject to some limitations. Since, the respondents answered questions with respect to their understanding, experiences and perceptions, it is possible that the collected data might not appropriately reflect the objectives of this study. Nonetheless, the quality of the data collected is satisfactory with regards to the nature of the report. Besides, the subjects were voluntarily recruited for this survey, albeit the believe that this did not negatively affect the findings since this approach has been usually employed in this field [29].

## Conclusions

The application of the research model demonstrated that nurses’ adoption of HEIMS in terms of the UB was influenced by SI and BI. Besides, SI and FC had the strongest influence on BI (serving as mediator) of UB to adopt and use HEIMS among the nurses in Ghanaian hospitals. Based on these findings, hospital administrators should put measures in place to ensure a conducive working environment for the nurses to continue using HEIMS as well as encourage and support them to adopt and use the system. Hospital managers should also continue to provide the necessary IT resources needed to use the HEIMS. These study results provide empirical and scientific contributions to aid better understanding of the behavioral issues regarding the readiness of nurses in the adoption of HEIMS in Ghanaian Hospitals. The outcome of this study will enrich the literature in this area and create an important awareness which will initiate further research, especially in developing countries.

## Data Availability

The datasets used and/or analysed during the current study are available from the corresponding author on reasonable request.

## Abbreviations

BI: Behavioral Intentions
FC: Facilitating Conditions
HAMS: Hospital Administration Management System
HEIMS: Hospital Electronic Information Management Systems
HERs: Electronic Health Records
HITs: Health Information Technologies
LHIMS: Lightwave Health Information Management System
SEM: Structural Equation Modelling
SI: Social influence
SPSS: Statistical Package for the Social Sciences
UB: Use Behavior
UTAUT: Unified Theory of Acceptance and Use of Technology
